# Hospital and Institutional News

**Published:** 1912-12-21

**Authors:** 


### HOSPITAL AND INSTITUTIONAL NEWS.

### THE SPEAKER AT THE KING'S FUND MEETING.

Owing to the full report of the proceedings at the annual meeting of the King's Fund on Wednesday last not coming to hand till we had begun to go to press, the list of awards has had to be held over until next week. We have, however, published the speech made by the Speaker of the House of Commons, which, owing to its importance, is given on another page. It is of great interest because it indicates certain important effects of National Insurance upon the voluntary hospitals which demand the earnest attention of Mr. Lloyd George, and will receive full consideration by voluntary hospital managers.

### CHRISTMAS IN MODERN ENGLAND.

What does Christmas really stand for in modern England? It is certainly worth while to ask, as inquiries show that there are at least three broad divisions of opinion on the point. There is the view hallowed by tradition of Christmas as the great festival of Churchmen; there is the traditional lay view, which partly conceals the former, of Christmas as a time of feasting; and there is the Puritan view tersely put by Mr. Bernard Shaw, who regards it as "a gluttonous spendthrift orgy foisted on us by unfortunate tradesmen who can just make both ends meet by the profits of the Christmas trade." This last is a reaction against the excess for which Christmas like other holidays has served as an excuse among temulent laymen. Since nobody can stand more than a little excess at any season, there have always been found those who insisted on the vulgar aspect of the festival, which is one that the bulk of the population, recruited by the eupeptic young, inevitably throws into the limelight. There is, too, the power of convention, the tyranny of habit, which makes many grown-up persons keep the festival, not because they enjoy the bustle, the presents, and the feasting, but on the ground that the children like it, as they certainly do. To hospital workers Christmas means first of all a large increase of work, but that being voluntarily done for others who expect it and are too ill to perform it for themselves has the reward of all super-selfish endeavour. That is the real reason why the singing, the clapping, and the trombone are welcomed by the sick people in hospitals to the surprise of every sensitive visitor who at once imagines the noise overdone. It is not precisely that it is enjoyed, except by the convalescents; but it is expected, and here is the secret really of the popularity of the plum-pudding to-day. Setting aside all vamped-up enthusiasm, and all the economic pressure of the butchers and shopmen at this time, the nation, whether it really enjoys the feast or not, expects it, and science has taught us that a striking change of habit is more irksome to the organism than the discomfort of an unwise practice or the penalty that besets a mistake. The spider respins a torn web from the first strand to the last because it finds routine less troublesome than foresight. And at the bottom of every grumble at Christmas is the instinctive desire to repeat the enjoyments that have been the mark of this season for nineteen hundred years.

### MR. GEORGE AT THE AGE OF ONE.

On Monday last Dr. George Griffiths, a well-known Welsh doctor and formerly chairman of the Pembrokeshire County Council, died at Bunker's Hill. It is not, however, on his medical attainments only or on his services as a county councillor that his claims to fame may rest. The deed of his life most likely to appeal to the popular imagination, of the present generation at any rate, is that he saved Mr. Lloyd George from an early and untimely death. At the age of one, the story is related, the future Chancellor was lying unconscious at a farmhouse suffering from croup and in urgent need of medical aid. It was a winter night, "the owl for all his feathers was a-cold," when news was conveyed to the late Dr. Griffiths of the plight of the unhappy child. Whether foreseeing in the depths of his unconsciousness the future success destined for his infant patient is not known, but Dr. Griffiths responded to the late call, made the journey, and succeeded in relieving the attack of croup. And at that time Mr. Lloyd George had none of his present claims to special consideration from the medical profession.

### SERIOUS CRITICISMS OF BRIDLINGTON WORKHOUSE INFIRMARY.

Severe strictures have been made on the condition of the Bridlington Workhouse Infirmary by Miss M. K. Lea, who at the request of the Local Government Board inspector has visited the institution and made a report. In it the ventilation and the mattresses were condemned as insufficient and "unfit for hospital use." The kitchen was described as "disgracefully dirty," and the nursing arrangements as unsatisfactory. These strictures the medical officer, Dr. W. A. Wetman, in the main confirms, except in so far as they reflect on the attitude of the nurse to the patients. "With the greater part of the report," writes Dr. Wetman to the Guardians, "I agree . . . the chief defects noted are due to the inadequacy of the present establishment, which makes it impossible to accommodate properly the inmates or increase the personnel of the staff. . . . The whole workhouse is nearly an infirmary, and the worst cases only are relegated to the hospital which has not even accommodation for this limited number, and is so constructed that it is impossible for one nurse to keep an eye on everybody. . . . As to defective bedding, the nurse is not responsible for that, and opinions differ as to when it should be condemned. As to dirty kitchens and closets, the help available is scanty, and the time of day at which the inspection was made (10 A.M.) should be borne in mind."

### THE NEED FOR ROOT AND BRANCH REFORM.

The Guardians have forwarded a letter in reply to the district inspector, stating that two meetings have been held, admitting much and palliating more. Their practical proposals may be judged from what they state as to the condition of the mattresses which was so severely complained of, and of which the medical officer could only say that "opinions differ as to when they should be condemned." "Some of the mattresses," the Board remarks, "are old and some stained, but the committee are of opinion that they are not in such a condition as to justify their being condemned, and they are quite capable of being cleaned and repaired." To the hospital expert the medical officer's letter above quoted is sufficient to condemn the institution and its administration outright. No matter what excuses may be adduced or fear of incurring expenditure be pleaded, it is evident on the facts stated that Bridlington Workhouse Infirmary is a disgrace to Poor-Law institutions as a class. As the medical officer hints, the slovenly administration is partly due to obsolete buildings and partly to that want of co-operation which always bolsters up abuses where infirmary and workhouse are administratively combined. Here, again, is a convincing proof that the complete separation of the infirmary from the workhouse is essential, even in a small infirmary like this, where the beds number eighteen only, though the medical officer states that only the worst cases are relegated there. We trust that instant measures will be taken to reform the administration and to reconstruct the institution root and branch.

### DEATH OF A LONDON HOSPITAL SECRETARY.

A well-known figure has been lost to the hospital world this week by the death of Mr. J. Francis Pink, late secretary of the Royal Dental Hospital, Leicester Square, and formerly secretary and librarian of Charing Cross Hospital Medical School. Until comparatively recently he successfully combined the duties at the medical school and the Dental Hospital, a plan which worked well, seeing that many of the dental students were also attached to Charing Cross Hospital. When the latter school was reorganised he remained on at the Royal Dental Hospital until a year or so ago, when he had to undergo two serious operations. Through Mr. Pink's hands have passed a very large number of medical and dental students, and well indeed did he look after them! His office at Charing Cross Hospital Medical School adjoined the library, and no small part of his duties used to consist in maintaining a serene and soothing atmosphere in his territory. His frequent and sudden incursions to quell a rising storm amongst the junior students were some of the most joyous incidents in the students' grind of those past days. The successive deans of both schools found in Mr. Pink something far more than a secretary; his watchful eye was over all, and not only did he extract belated fees with exceptional and painful success, but he bestowed an almost fatherly care on the students throughout their school career. Many are the men who to-day owe much to his kindly and practical interest. In addition he was able to act as local registrar. Mr. Pink never completely regained strength after his operation, and was in bad health for some months before his death. He was in his sixty-ninth year. One of his sons forsook dental surgery in favour of the stage.

### SIR G. NEWMAN ON A NATIONAL EVIL.

From the welter of controversy which the medical inspection of schools has created it is instructive to turn for a moment to the Report of Sir George Newman, Chief Medical Officer of the Board of Education, where may be found a practical summary of all the facts revealed so far concerning the health and general condition of elementary scholars by legislation. To the examination on entrance and at leaving an intermediate inspection is likely to be added. Since the passing of the Act about one million and a half children have been dealt with annually, and the general result is the revelation of a deplorable amount of malnutrition and its consequences in the up-growing generation. Good nutrition could be affirmed of only 45 per cent. at Burton among all the children in thirteen counties and sixteen urban areas. Brighton comes next with 41 per cent., and below the proportions vary from 26.0 to 3.8. At first glance, the evil does not necessarily seem confined to industrial districts, for Gloucestershire has the low figure of 5.2, although Middlesex figures lowest with 3.8 only. It must also be remembered, as we have only recently pointed out in comparing the figures of American and English clinics, that the absence of an absolute standard leads to individual estimates being put forward that depend very largely on the bias of individual inspectors. In short, were the inspectors transferred to different districts the figures next adduced would tend to vary accordingly. Still, taking a mean standard for the epithet "good," the general average is a horribly low one. As to the feeble-minded now attracting attention, Sir George Newman estimates the numbers of this rather loosely defined section as 27,000 in England and Wales, or about 1½ per cent. The causes are that well-known array of civilised plague spots, overcrowding, malnutrition, and the rest, together with the ignorance which flourishes in their midst. As our "Maternity and Mothering" articles have already demonstrated, this ignorance may be illustrated by the ubiquity of the baby's "comforter"—a scourge which the Registrar-General's Returns seem to show more productive of death than any actual disease. The diagnosis of disease among school children has revealed a national evil. Expense or no expense the schools will have eventually to follow up their investigations with preventive measures.

### A VETERAN CONSUMPTION SPECIALIST.

The Brompton Hospital and the London medical profession have again to deplore the death of a distinguished veteran. Hard upon the decease of Dr. Reginald Thompson a few weeks ago comes that of his old colleague, Dr. Theodore Williams, at the age of seventy-four years. Dr. Williams was the son of Dr. C. J. B. Williams, Physician Extraordinary to Queen Victoria, who died at an advanced age only a few years ago. His early career at Oxford and at St. George's Hospital was one of great promise, duly fulfilled in later life as physician to the Brompton Hospital for Consumption, and as member and president of medical societies too numerous to be recited individually. He was always distinguished for the generosity with which he would help any deserving professional institution or individual. His college at Oxford, Pembroke, elected him to an Honorary Fellowship in 1907, in recognition of his liberality in founding medical scholarships there; and his services to the study of tuberculosis and to the King Edward VII. Sanatorium as physician were recognised in 1906 by the bestowal of the Victorian Order. Nor were Dr. Williams' activities restricted to professional affairs; he exhibited a wide culture which embraced many subjects, and was in fact a gentleman and a scholar as well as a man of the world and a physician of ripe experience and knowledge.

### THE ECONOMICS OF FINE ART.

The Windsor Castle State Apartments Fund has enabled the King to forward the following sums to local hospitals and charities: King Edward VII. Hospital, Windsor, receives £500; St. Andrew's Hospital, Clewer, £50; and the Slough Nursing Fund, £40. How many of the visitors who have passed through this royal turnstile, which regularly clicks out money for the hospitals, paused to think of the strange light on the economics of fine art which their visits give? For the consumption of objects of art differs from that of every other commodity in that the more they are consumed the more they produce, and the larger the number of those whose pleasure they serve the more pleasure they have to distribute. The institutions, however, which benefit by the funds of Windsor, Chatsworth, and other museums of art treasures may well take to heart the tacit lesson in economics which accompanies the cheque.

### ELECTRICITY AND ITS RISKS.

The case in which Dr. Hall-Edwards was recently sued for damages by a Birmingham merchant, who claimed that the negligent use of an electric current had set up eczema of nine months' duration, is of interest from several points of view. In the first place, it serves to emphasise the lessons of the fatal case on which we commented a month ago (The Hospital, November 16, 1912, p. 182), and to show the necessity of close expert supervision whenever these powerful methods of electro-therapy are selected for application to the human body. Next, it is interesting because the current employed was apparently the not long introduced method of diathermy, a description of which appeared in our columns as far back as February 10 last. It was noted at the time that this invention enables enormous currents to be safely used; but it is clear that the method, valuable as it is for many cases, needs discretion in its application. Next, the personality of the defendant adds interest to the case. Dr. Hall-Edwards is one of those early pioneers of *x*-ray work who suffered slow martyrdom for their researches; and has received a pension from the Government in recognition of his services to science and their unfortunate consequence. Lastly, the case is important for what it did not reveal as well as what it did. The defence made an effort to bring out that the complaint for which the electrical treatment was sought was locomotor ataxia; but were estopped from doing so by the plaintiff's lawyers on technical grounds. This unwillingness to have the matter thrashed out, together with the lack of any disclaimer from the plaintiff, indicates a strong probability that locomotor ataxia was in fact the latter's disease. If that were so, it is easy to understand that a burn or a dermatitis might easily arise quite apart from any negligence; and it may serve as a caution against the indiscriminate use of diathermic currents for those who suffer from any nervous disease in which disturbances of cutaneous innervation are a feature. Lastly, it is to be noted that the Medical Defence Union have added one more to their laurels; this time, let us hope, they will be able to recover their costs, which so often are unobtainable in the cases which they successfully defend.

### A CALL TO PROVINCIAL HOSPITALS.

The scientific standing and resources of the provincial hospitals have never been better recognised by the Metropolis than in a recent decision of the directors of the coming International Congress of Medicine to form an Illustrative Museum in London of scientific materials from provincial hospitals. It is felt that the sections of the Congress would gain very largely by the formation of an Illustrative Museum to supplement them, and a committee has been formed, with Professor Arthur Keith, conservator of the Royal College of Surgeons' Museum, to organise it. The Museum will be established at the Imperial College of Science at South Kensington, and as the visitors to the Congress will naturally wish to inspect the Museums of the London Hospitals, the happy idea has been formed of inviting exhibits from provincial institutions. The specimens, it should hardly be necessary to add, will exhibit the scientific to the exclusion of the commercial side of medicine. If the provincial hospitals rally to this call, the international visitors will have a unique opportunity of observing the massed material which the hospitals of the whole country have collected up to the present time. The provincial hospitals, by lending their scientific treasures, will be represented in London, with no possibility of their contributions to the Museum being confused with those of their sister institutions in London.

### POSSIBLE SITES FOR THE LISTER STATUE.

The subscription lists published in the press show that the appeal for funds to establish a Lister Memorial has met with a hearty response. The triple nature of the Memorial, doubtless, has something to do with this cordial support, for we can scarcely think that such generous donations would have come in wholly in support of the proposal to erect a statue to the memory of the great surgeon.

As a matter of fact, the suggestion of a statue is only too likely to debar would-be sympathisers from subscribing until they know where the effigy is to be placed, who is to fashion it, and what form it will take. These are questions which the Memorial Committee should take the earliest opportunity of answering. The first is, perhaps, the most important. There are two hospitals in London that are specially identified with the name of Lister—University, where he studied, and King's, where much of his best work was done. But the new University College has only sentimental associations with his name, while the new King's, which is rising towards completion at Denmark Hill, never knew the master. We would venture to suggest that the site of the old King's College Hospital is one admirably suited for a statue of Lister. When the old buildings are dismantled it would be comparatively easy to secure a vacant space for the statue in a position which would be central and commanding. Failing that, the magnificent approach to the new back of the British Museum would be another suitable site. In any case, we hope that the Memorial Committee will take the public into its confidence and state what other positions, if any, are under consideration.

### THE NEW CITY MEDICAL OFFICER.

Dr. William James Howarth, medical officer of the county of Kent, has been elected medical officer of the City in succession to Dr. William Collingridge, with whom we published an interview at the time when his retirement was announced. Dr. Howarth, who was born in 1868, studied at Manchester University, where he graduated in medicine and surgery. His experience of public health work has been continuous for fifteen years, his previous appointments including those of medical officer for Derbyshire, which he held for ten years, and medical officer for Kent, where he has been for nearly five years past. Dr. Howarth is president of the Home Counties Branch of the Incorporated Society of Medical Officers of Health and a major of the Home Counties Division of the Territorials. Apart from Dr. Howarth, the remaining two of the three finally selected candidates for the post of medical officer of the City were Dr. W. M. Willoughby, senior assistant medical officer of the Port of London, and Dr. R. A. Lyster, medical officer of the county of Hampshire.

### THE BEIT RESEARCH FELLOWSHIPS.

The list of those elected to Beit Memorial Fellowships at the meeting of the Trustees held upon December 13 is interesting as showing the cosmopolitan tendencies of science and of scientific medicine. There are ten Fellows on the list, three of whom are women. Two of the ten are *alumni* of Cambridge University; there is one graduate of Oxford, and one of London University. The remaining six have been educated at Sydney, Berne, Toronto, Aberdeen, Melbourne, and Würzburg; two of them are foreigners, and three are Colonials. Only one of the Fellows proposes to carry out his researches abroad, however, and that is a Canadian who proposes to study in Munich. Although in one way it is natural to feel a faint regret that so many of these endowments should go to others than Englishmen; on the other hand, it is matter for congratulation that our Colonies are providing so many capable recruits to science. There is something especially appropriate in this, inasmuch as Beit himself (who was not an Englishman by race, nor even a European) made a large part of his colossal fortune out of British Colonies. With three-tenths of the total appointments, the women have got their full share, since this proportion far exceeds that of their numerical strength as compared with male practitioners.

### LAZINESS DISGUISED AS CHARITY.

A curious summons against a practitioner was heard at Bow Street last Saturday. Dr. Alfred Benson, the medical defendant, was charged by the Director of Public Prosecutions with knowingly and falsely signing two declarations vouching personal knowledge that two men were fit and proper persons to receive passports to travel to Rotterdam. In support of the summons Mr. Bodkin said that the men in question applied for passports on April 10, producing the usual declarations, which were signed by the defendant. The passports were duly granted, and the next that was heard of the two men was that they had been arrested in Strasburg for procuration. There was no suggestion that the defendant knew that the men were engaged in the white-slave traffic. The Magistrate committed the defendant for trial, allowing bail in £100. Whatever be the upshot of the case, the profession in particular, to which an ever-increasing number of forms and declarations are being submitted, and the public generally, should learn to use the greatest caution in signing declarations of this class. Still, the moral is that no one, whether a doctor or anyone else, has any moral or legal business to sign a declaration of good character for a person he knows nothing about; and when that declaration is for the information of the Foreign Office in connection with the grant of a passport, this obligation carries extra cogency. Not very long ago a doctor was fined for signing a passport declaration about a person with whom he was not properly acquainted. Why doctors should distinguish themselves by laziness disguised as charity in this matter is difficult to say. It is to be hoped that this will be the last charge to be brought against the profession.

### THE COUNTY COUNCIL'S PLANS FOR CONSUMPTIVES.

Temporary provision of sanatorium accommodation for insured tuberculosis persons has been arranged between the London County Council, the Metropolitan Asylums Board, and the London Insurance Committee. As soon as practicable the Board will provide accommodation for some 130 patients, to be increased annually until provision for about 500 is available. The Council will arrange with the Insurance Committee for the selection of the patients. The Insurance Committee agree to send patients to the sanatorium provided by the Council rather than to any other institution, and to keep on an average 400 patients there. The arrangement will continue in force until March 1914, and may afterwards be terminated at three months' notice. The Council will pay the Asylums Board the actual cost incurred by them in providing treatment for County Council patients. The Insurance Committee will pay the Council £1 15s. a week for each insured person treated, and a periodical adjustment will be made from time to time based upon the actual expenditure of the Council. The weakness of the scheme, for it has weaknesses, was pointed out in The Hospital last week.

### THE TRUE USE FOR THE WESTMINSTER SITE.

It is gratifying to learn from Mr. W. Benn, M.P., as representing the First Commissioner of Works, that the Government is considering how the site of Westminster Hospital, if the institution receives permission to move, can be used "in a manner suitable to the dignity of the situation." How this dignity can be best preserved there is no question. If the hospital moves the site should be cleared, and not built upon for any purpose whatsoever. London has far fewer open places than any capital of similar importance, and probably a more continuous tradition of wasted opportunities than them all. There is in Parliament Square and by Broad Sanctuary the nucleus of an open space that any Government with a sincere eye for improving the beauties of London would mark at once for future development. Paris has made itself the laughing-stock of Europe for finding no work for Rodin; let us hope that, while our opportunities are not so magnificent, we may not follow the Parisian precedent for neglecting them.

### TUBERCULOSIS DISPENSARIES IN LONDON.

The Local Government Board has made certain suggestions to the Public Health Committee of the London County Council for the permanent treatment of tuberculosis in London. Among them we note first that the unit should be the Metropolitan Borough, and Borough Councils have been invited to form these institutions. The County Council is invited to formulate a complete scheme for the Capital, for which purpose a central organising body for the provision of institutional treatment, whether indoor or outdoor, is suggested. If the Council will hold itself responsible for the provision of in-patient accommodation the grant in aid of this accommodation will be paid to it. In the opinion of the Public Health Committee the Council should await comprehensive reports from the committees concerned before it expresses its views on the matter. These reports alone can dissipate the vagueness that enfolds the Board's proposals at the present stage.

### PURE MILK AS AN IDEAL.

The Milk and Dairies Bill, which Mr. John Burns, with Mr. Herbert Lewis, has introduced for the registration and inspection of dairies, has already received criticism on the ground that, if inspection does not cover the whole transit of milk from the udder of the cow to the mouth of the consumer, it is useless. How useless the exploiter of this criticism proceeded to show by an account of the handling of milk and cans, which his own eyes witnessed, by porters at one of the London railway stations. The criticism is just in so far as it relates to the necessity for supervision of the means of transit, but, on the other hand, it must be remembered that, when every care which Parliament can devise and even enforce has been bestowed on the cow, the dairy, and the railway porter, milk has an enemy with which Parliament is powerless to cope—namely, the actual consumer. Probably quite as much milk is contaminated in the consumers' premises as in the dairy and in the railway trains. While this emphatically is not an argument for the inspection of private premises, still less for the abandonment of all reasonable inspection and care, it points to the common-sense fact that perfect purity of milk is as impossible for England, as perfect security on sea, or absolute asepsis in hospitals. We recently recalled the elaborate ritual that once obtained in German hospital theatres, and which ended in becoming an intolerable parade that produced no better result than less elaboration. Reasonable care is all that can or should be insisted on; pure milk, in an absolute sense, is neither possible, nor perhaps worth such elaborate precautions as the inspection of consumers' premises would imply.

### THE DOCTORS AND THE CHANCELLOR.

The exact reply of the British Medical Association to the Chancellor of the Exchequer cannot be known until next week. Nevertheless the votes, as recorded in the many districts which have already considered the matter, enable it to be said with fair accuracy that there is a majority of about five to one in favour of refusing the terms offered. If the minority could be relied upon to stick to the Association, and abide loyally by the result of the voting, the position of the doctors would be a very strong one. But it is abundantly clear that nothing of the sort can be expected; and an amazed public has this week seen Dr. Lauriston Shaw, actually the chairman of the Medico-Ethical Committee of the Association, doing his best to wreck the Association by repudiating his own pledges and advising others to do the same. Thus it can be predicted that Mr. George will obtain, not indeed enough practitioners to serve the whole of the insured, but enough seriously to weaken the Association and to set up panels in a few districts. In these circumstances, we question whether it is good tactics to continue the struggle. We believe that cohesion in the profession now would involve the Chancellor's capitulation to their reasonable and just demands; but since that cohesion cannot be relied upon, we doubt if it will do any good to split the Association. It will be interesting to see if appointments under Government or under the Act are bestowed upon Drs. Addison, Mills, and Shaw, the brains of the threatened secession. For no one doubts that their disloyalty to their profession will meet with some adequate expression of Mr. Lloyd George's gratitude.

### GREAT GIFT FOR THE NEW WOMEN'S HOSPITAL.

The proposed scheme of a South London Hospital for Women has entered on a new and startling phase by the announcement made last Tuesday at a drawing-room meeting, over which Lady Bertha Dawkins presided, that some anonymous ladies are now offering a site at Clapham and £25,000 towards the cost of the hospital. At the same meeting Dr. Helen Webb stated that a site had been acquired at Clapham Common, but that the out-patient department would be at Newington Causeway. These latter statements, however, are not news to readers of The Hospital, for whom the scheme was summarised early in September, and its bearings discussed in a subsequent article. There can be no doubt, however, that the new gift of £25,000 will assist the early maturity of the scheme, though among the rumours afloat is one that the anonymous givers were responsible for the Clapham site from the first. In any case the present announcement will strengthen the hands of Doctress Davies-Colley and the committee.

### THE HOSPITAL DOLL SHOW.

On Wednesday and Thursday this week, as we announced to take place in a recent issue, the thirty-third *Truth* Doll Show, which has given for a generation toys to children in Poor-Law infirmaries, hospitals, workhouses, and schools, was held at the Albert Hall. The Queen headed the nation's givers with a national group of three large dolls, christened "Rose," "Shamrock," and "Thistle," and in addition to six thousand dolls on show there were twenty thousand small toys available for distribution. The large dolls, which were over one hundred in number, are to be given for the common use of patients in the various institutions, and, indeed, the Queen's dolls, which were 42 inches in height, are too large as "babies" to be nursed by "mothers" about their own size. The Sir Francis Tress Barry Fund, which gives a sixpence to each elder child in the hospitals and workhouses, was represented by a basket containing 7,000 new sixpenny pieces. As we emphasised before, hospital and institutional workers owe a special debt of loyalty and gratitude to *Truth*, which was the first newspaper to invent the idea of a toy show for hospital children, and the first to show how such a vast undertaking could be organised with success. We trust many hospital workers recognised this, first by dressing dolls or sending subscriptions to the toy fund; and, secondly, by attending the exhibition at the Albert Hall, for the *Truth* Doll Show is an annual hospital and infirmary festival.

### EGYPT AND THE WAR FUNDS.

The extraordinary enthusiasm manifested on behalf of the Red Cross and Red Crescent movements throughout Europe is well exemplified in the great success of the Red Crescent and Turkish War Fund in Egypt. According to the Cairo correspondent of the *Times* a total of £110,000 has been subscribed up to date, and one of the chief factors in the success of this fund appears to have been the tour made by Prince Mohamed Ali in Upper Egypt, from which the bulk of the money has been derived. Egypt, no doubt, has a primary interest in the struggle from the fact that the Turkish soldiers are co-religionists of the principal section of the population, but it is interesting to correct our British prejudice by observing that the Mansion House alone has no necessary monopoly of power to raise funds which run into six figures.

### THE SALE OF MEDICATED WINES.

Before the Select Committee on Proprietary Medicines adjourned for the Christmas Vacation, evidence was given by Mr. H. J. Hall, the founder and managing director of Stephen Smith and Co., Limited, proprietors of Hall's Wine. He said that the sale was influenced by the recommendation of doctors who had taken the wine themselves, and had been so satisfied with it that they had recommended it to their patients. The original idea to put the wine on the market, he stated, came from a medical man, and the medical profession had always been advised as to its method of preparation; it contained port wine and extracts of meat and malt. Asked by the Chairman if Stephen Smith was a doctor the witness replied that he was a Member of the Royal College of Surgeons. Replying to a further question he said that Hall's Wine contained one grain of extracted principles of the coca-leaf in a bottle of 26 ounces.

### THE STIMULATION OF BACKWARD BABIES.

Under the ægis of an illustrated daily newspaper a campaign is about to be started having for its objects the stimulation of backward babies and the circulation of our contemporary. The scheme includes the distribution of a thousand pounds in prizes and of daily doses of instruction in baby culture in the columns of the newspaper in question. The medical profession has been circularised and provided with certificates to hand over to the mothers, and presumably to afford the doctors a chance of gaining a humble fee by filling up a delightfully vague report—or will they do it by force of habit for nothing? With the aid of these forms, and without seeing the babies, a doctor in London will select the "most perfect" babies as best he can, and then nine practitioners in various parts of the United Kingdom will somehow and somewhere regard the infants face to face preparatory to the prize-giving. We gather that the guiding principle is to be "fitness, not fatness," and we wish the gentleman who is to select the perfect-on-paper babies every joy while he is assessing the medical certificates. One little point seems to have been overlooked, and that is at what age is a baby no longer a baby.

### THE DRUG MARKET IN HOLIDAY MOOD.

With the approach of Christmas business has begun to assume a holiday mood, and, apart from covering their immediate requirements in the way of drugs, buyers are not disposed to operate freely. Few changes in price have taken place during the week. Opium is quiet and practically unchanged in price. Castoreum sold at lower prices at the recent public sales. Among articles which are dearer are Indian hemp, essence of lemon, orris-root, and phenacetin. A fair business has been done in eucalyptus oil, which is in seasonable demand.

